# In Vitro Neutralization of the Myotoxicity of Australian Mulga Snake (*Pseudechis australis*) and Sri Lankan Russell’s Viper (*Daboia russelii*) Venoms by Australian and Indian Polyvalent Antivenoms

**DOI:** 10.3390/toxins14050302

**Published:** 2022-04-26

**Authors:** Prabhani Thakshila, Wayne C. Hodgson, Geoffrey K. Isbister, Anjana Silva

**Affiliations:** 1 Department of Parasitology, Faculty of Medicine and Allied Sciences, Rajarata University of Sri Lanka, Anuradhapura 50008, Sri Lanka; prabhanithakshila123@gmail.com; 2 South Asian Clinical Toxicology Research Collaboration (SACTRC), Faculty of Medicine, University of Peradeniya, Peradeniya 20400, Sri Lanka; geoff.isbister@gmail.com; 3 Monash Venom Group, Department of Pharmacology, Biomedical Discovery Institute, Monash University, Clayton, VIC 3800, Australia; wayne.hodgson@monash.edu; 4 Clinical Toxicology Research Group, University of Newcastle, Newcastle, NSW 2298, Australia

**Keywords:** myotoxicity, efficacy, antivenom, *Daboia russelii*, *Pseudechis australis*

## Abstract

We studied the neutralisation of Sri Lankan Russell’s viper (*Daboia russelii*) and Australian mulga snake (*Pseudechis australis*) venom-induced myotoxicity by Indian (Vins and Bharat) and Australian (Seqirus) polyvalent antivenoms, using the in vitro chick biventer skeletal muscle preparation. Prior addition of Bharat or Vins antivenoms abolished *D. russelii* venom (30 µg/mL)-mediated inhibition of direct twitches, while Australian polyvalent antivenom was not protective. Bharat antivenom prevented, while Vins and Australian polyvalent antivenoms partially prevented, the inhibition of responses to exogenous KCl. Myotoxicity of Mulga venom (10 µg/mL) was fully neutralised by the prior addition of Australian polyvalent antivenom, partially neutralised by Vins antivenom but not by Bharat antivenom. Although the myotoxicity of both venoms was partially prevented by homologous antivenoms when added 5 min after the venom, with an increasing time delay between venom and antivenom, the reversal of myotoxicity gradually decreased. However, antivenoms partially prevented myotoxicity even 60 min after venom. The effect of antivenoms on already initiated myotoxicity was comparable to physical removal of the toxins by washing the bath at similar time points, indicating that the action of the antivenoms on myotoxicity is likely to be due to trapping the toxins or steric hindrance within the circulation, not allowing the toxins to reach target sites in muscles.

## 1. Introduction

Snakebite causes considerable morbidity and mortality due to the acute effects of the venom, and, less often, the long-term sequelae of venom effects. Local necrosis, venom-induced consumption coagulopathy, neuromuscular paralysis, acute kidney injury and myotoxicity are common, clinically important, acute effects of snake envenoming [1]. Antivenoms remain the only specific treatment available for snakebite as they have been for over a century [2].

Myotoxicity, or venom-induced muscle damage, is less well understood compared to other acute effects of envenoming and is relatively common after bites by some Australian elapid snakes and sea snakes [3]. Some viperid and elapid snake envenomings result in local and systemic muscle injury, manifesting as local and generalised myalgia, muscle tenderness, trismus, myoglobinuria, and severe rhabdomyolysis [3,4]. Phospholipases A_2_ (PLA_2_) toxins are the most abundant myotoxic components in viper and elapid venoms [3,5]. Most myotoxic viperid venoms only induce local muscle injury, with a few exceptions, including the South American rattlesnake (*Crotalus durissus terrificus*) and Sri Lankan Russell’s viper (*Daboia russelii*), inducing systemic muscle injury. While some Australian elapid venoms induce systemic myotoxicity [3,4,6].

Sri Lankan Russell’s viper envenoming generally causes mild systemic myotoxicity in humans [4]. Two synergistically acting weakly myotoxic PLA_2_ toxins, i.e., U1-viperitoxin-Dr1a and U1-viperitoxin-Dr1b, in Russell’s viper venom are responsible for this mild myotoxicity [4]. It has been shown that, despite Sri Lankan Russell’s viper venom being weakly myotoxic, Indian polyvalent antivenom (Vins bioproducts) was unable to neutralise the in vitro myotoxicity in the chick-biventer nerve-muscle preparation [4]. In contrast to Sri Lankan Russell’s viper venom, Australian mulga snake (*Pseudechis australis*) venom contains potent myotoxins, including mulgatoxin-a [7], and bites by this species can result in severe systemic myotoxicity in some patients. Indeed, peak creatine kinase concentrations exceeding 100,000 U/L are reported in some patients [3,8]. Although antivenom did not reverse the already established myotoxicity, early antivenom within 2 h, appeared to prevent myotoxicity in three patients who had high venom concentrations in their pre-antivenom blood samples [8]. This was in agreement with previous observations made in in vivo anaesthetised rats, in which administration of antivenom one-hour post-envenoming resulted in a lower increase in plasma creatine kinase concentration compared to administration of antivenom six hours post-envenoming [9]. The efficacy of the Indian antivenom on the already initiated myotoxicity of Russell’s viper venom has not been experimentally studied in the chick-biventer model. However, the efficacy of black snake and tiger snake antivenoms added to the chick-biventer preparation one hour post-venom partially prevented the myotoxicity induced by Mulga snake venom [10].

Despite their structural and mechanistic differences, it has been hypothesised that PLA_2_ toxins cause myotoxicity by damaging the sarcolemma followed by a sequence of degenerative events in the muscle cells. This muscle injury recovers with regeneration [5,11]. Although antivenoms are used to treat myotoxicity in snake envenoming, the ability of antivenom to reverse already initiated venom-induced muscle injury requires further exploration.

This study aimed to investigate the comparative neutralisation of Sri Lankan Russell’s viper venom- and Australian mulga snake venom-induced myotoxicity by Indian and Australian polyvalent antivenoms, and to investigate the time windows during which the antivenoms remain capable of preventing the already initiated venom-induced muscle injury in vitro.

## 2. Results

### 2.1. In Vitro Myotoxicity Caused by D. russelii Venom

**Figure 1:**
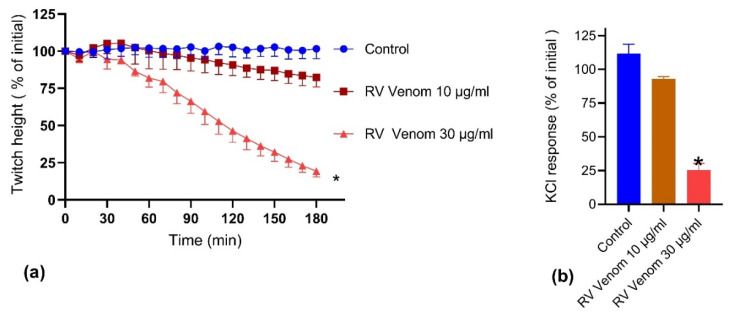
In vitro myotoxicity of *D. russelii* venom in the chick biventer nerve-muscle preparation: Effect of venom on (**a**) direct twitches (* significantly different from control at 180 min; *p* < 0.05, one-way ANOVA following by Tukey’s posthoc test, *n* = 3–5); and (**b**) contractile responses to KCl (40 mM) (* significantly different from the control at 180 min; *p* < 0.05; one-way ANOVA followed by Tukey’s posthoc test, *n* = 3–5).

Sri Lankan *Daboia russelii* venom (10 and 30 µg/mL) caused inhibition of direct twitches in the chick biventer nerve-muscle preparation (Figure 1a) and inhibited the contractile response to KCl 40 mM (Figure 1b), indicating skeletal muscle damage. *D. russelii* venom 30 µg/mL, which resulted in a 80% decrease in direct twitch height after 180 min, was selected for subsequent myotoxicity prevention and reversal experiments. *D. russelii* venom 10 µg/mL was only able to cause an 18% decrease of the direct twitch height after 180 min.

### 2.2. In Vitro Myotoxicity Caused by P. australis Venom

**Figure 2:**
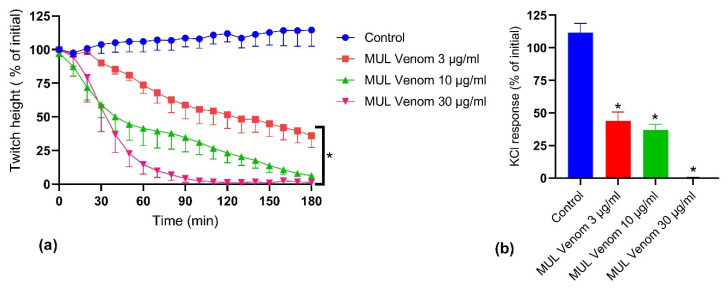
In vitro myotoxicity of *P. australis* venom on the chick biventer nerve-muscle preparation: Effect of venom on (**a**) direct twitches (* significantly different from control at 180 min; *p* < 0.05, one-way ANOVA followed by Tukey’s posthoc test, *n* = 3–5); and (**b**) contractile responses to KCl (40 mM) (* significantly different from the control at 180 min; *p* < 0.05, one-way ANOVA followed by Tukey’s posthoc test, *n* = 3–5).

Australian mulga snake (*Pseudechis australis*) venom (3, 10 and 30 µg/mL) caused inhibition of direct twitches in the chick biventer nerve-muscle preparation (Figure 2a) and inhibited the contractile response to KCl (40 mM; Figure 2b), indicating skeletal muscle damage. *P. australis* venom 10 µg/mL, which led to a 93% decrease in the direct twitch height after 180 min, was selected for subsequent myotoxicity prevention and reversal experiments.

### 2.3. Prevention of the In Vitro Myotoxicity Caused by D. russelii Venom by the Prior Addition of Antivenom (Pre-Venom Prevention Study)

**Figure 3:**
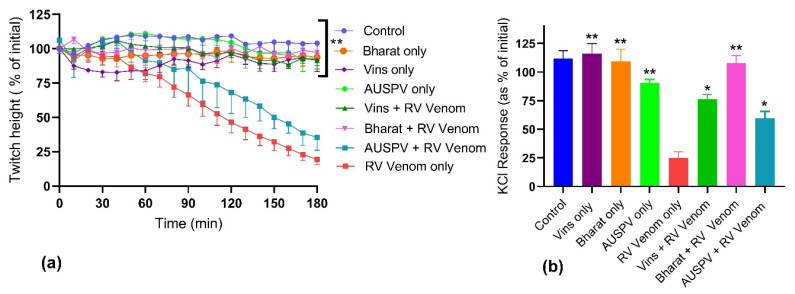
The effect of the prior addition of Vins, Bharat or Australian polyvalent (AUSPV) antivenoms on the in vitro myotoxicity of *D. russelii* venom (RV, 30 µg/mL) in the chick biventer nerve-muscle preparation: Effect on (**a**) direct twitches; and (**b**) on the response to 40 mM KCl (* significantly different from both control and venom,** significantly different from venom but not control; *p* < 0.05; One-way ANOVA following by Tukey’s posthoc test, *n* = 3–5).

Prior addition of either Bharat or Vins antivenoms, at the recommended concentrations, abolished *D. russelii* venom (30 µg/mL)-mediated inhibition of direct twitches in the chick biventer nerve-muscle preparation. In contrast, the prior addition of Australian polyvalent antivenom, at a concentration recommended for an equivalent quantity of *P. australis* venom, failed to prevent the inhibition of direct twitches induced by *D. russelii* venom (Figure 3a). In addition, Bharat antivenom prevented the inhibition of responses of the muscle to KCl, while Vins and Australian polyvalent antivenoms only partially prevented this inhibition (Figure 3b). The three antivenoms alone had no significant effect on direct twitches compared to control.

### 2.4. Prevention of the In Vitro Myotoxicity Caused by P. australis Venom by the Prior Addition of Antivenom (Pre-Venom Prevention Study)

**Figure 4:**
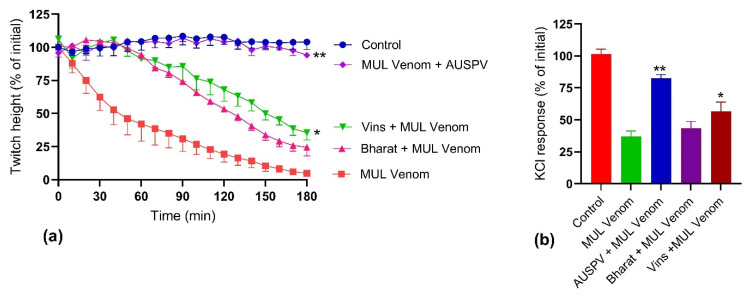
The effect of the prior addition of Australian (AUSPV), Vins or Bharat polyvalent antivenoms on the in vitro myotoxicity of *P. australis* venom (MUL venom, 10 µg/mL) in the chick biventer nerve-muscle preparation: Effect on (**a**) direct twitches; and (**b**) on the response to 40 mM KCl (* significantly different from both control and venom,** significantly different from venom but not control; *p* < 0.05; One-way ANOVA following by Tukey’s posthoc test, *n* = 3–5).

Prior addition of Australian polyvalent antivenom, at the recommended concentration, abolished *P. australis* venom (10 µg/mL)-mediated inhibition of direct twitches in the chick biventer nerve-muscle preparation (Figure 4a). In contrast, the prior addition of Vins antivenom, at a concentration recommended for an equivalent quantity of Russell’s viper venom, only partially inhibited the effects of *P. australis* venom. The prior addition of Bharat antivenom, at a concentration recommended for an equivalent quantity of Russell’s viper venom, had no significant effect on myotoxicity. Similar effects were observed for the antivenoms’ ability to prevent inhibition of contractile responses to KCl, with the Australian antivenom preventing inhibition, the Vins antivenom partially preventing inhibition and the Bharat antivenom having no significant effect (Figure 4b).

### 2.5. Post-Venom Prevention of D. russelii Venom-Mediated Myotoxicity by Indian Polyvalent Antivenoms In Vitro Compared to Washing

**Figure 5:**
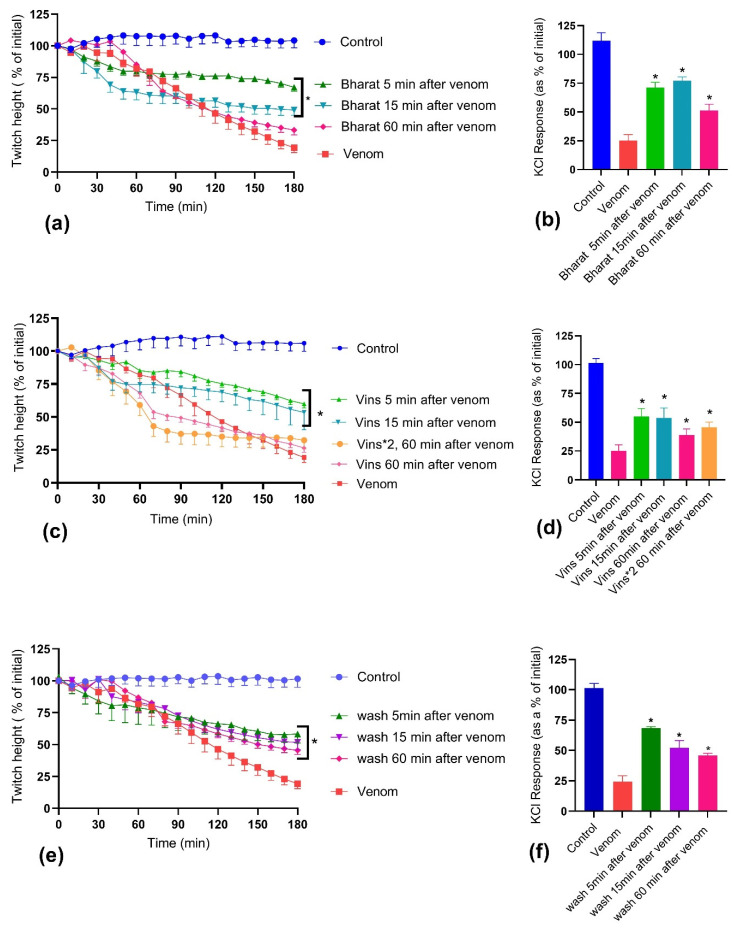
Effect of post venom addition of Vins antivenom, Bharat antivenom or washing, on *D. russelii* (10 µg/mL) venom-induced myotoxicity in the chick biventer nerve/muscle preparation. Effect on (**a**) inhibition of direct twitches and (**b**) inhibition of the contractile response to 40 mM KCl when Bharat antivenom was added 5, 15 and 60 min after the venom; on (**c**) inhibition of direct twitches and (**d**) inhibition of the contractile response to 40 mM KCl when Vins antivenom, at the recommended concentration, was added 5, 15 and 60 min after the venom and when two times the recommended concentration of antivenom was added after 60 min; on (**e**) inhibition of direct twitches and (**f**) inhibition of the contractile response to 40 mM KCl when the bath was washed with physiological salt solution 5, 15 and 60 min after the venom (* significantly different from both control and venom; One-way ANOVA following by Tukey’s posthoc test, *n* = 3–5).

Bharat and Vins antivenoms, at manufacturer-recommended concentrations, partially prevented the 30 µg/mL *D. russelii* venom-mediated inhibition of direct twitches (Figure 5a,c) of the chick-biventer nerve-muscle preparation, as well as the venom-mediated inhibition of the contractile response to 40 mM KCl (Figure 5b,d), when added to the bath 5 and 15 min after venom. Neither antivenom, when added 60 min after venom, had a significant effect on the inhibition caused by 30 µg/mL *D. russelii* venom (Figure 5a,c). However, both antivenoms partially prevented the venom-mediated inhibition of the contractile response to 40 mM KCl (Figure 5b,d). Even two times the recommended quantity of Vins antivenom, added 60 min after venom, failed to prevent the myotoxicity (Figure 5c,d).

The physical removal of the venom, by washing the bath with the physiological salt solution 5, 15 and 60 min after adding the venom, partially prevented 30 µg/mL *D. russelii* venom-mediated inhibition of direct twitches (Figure 5e) of the chick-biventer nerve-muscle preparation, as well as the venom-mediated inhibition of the contractile response to 40 mM KCl (Figure 5f).

### 2.6. Post-Venom Prevention of P. australis Venom-Mediated Muscle Injury by Indian Polyvalent Antivenoms In Vitro Compared to Washing the Bath

**Figure 6:**
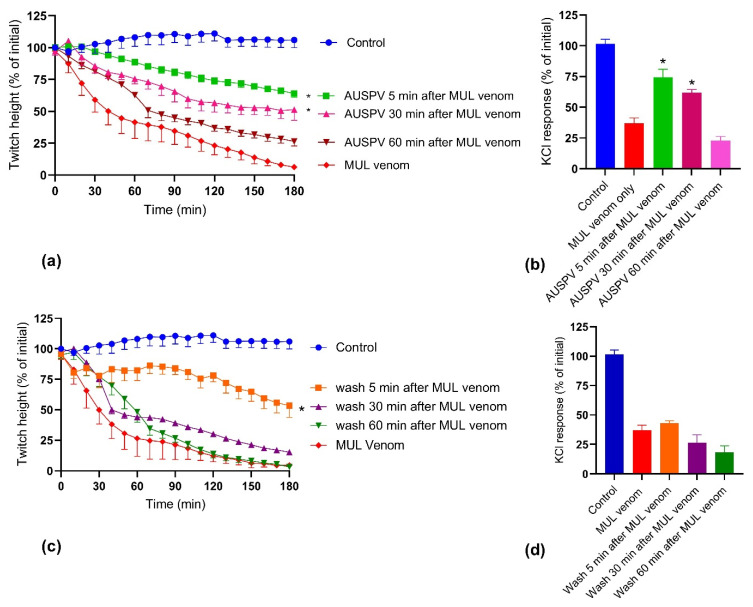
Effect of Australian polyvalent antivenom (AUSPV), or washing, on the post-venom prevention of *P. australis* (MUL) venom-induced myotoxicity in the chick biventer nerve/muscle preparation: reversibility of (**a**) inhibition of direct twitches; or (**b**) inhibition of the contractile response to 40 mM KCl when Australian polyvalent antivenom was added 5, 30 and 60 min after the venom; reversibility of (**c**) inhibition of direct twitches and (**d**) inhibition of the contractile response to 40 mM KCl when the bath was washed with the physiological salt solution 5, 30 and 60 min after the venom (* significantly different from both control and venom; One-way ANOVA following by Tukey’s posthoc test, *n* = 3–5).

Australian polyvalent antivenom, at the recommended concentration, partially prevented 10 µg/mL *P. australis* venom-mediated inhibition of direct twitches (Figure 6a) when added to the bath 5 min and 30 min after the venom. In addition, Australian polyvalent antivenom partially prevented the inhibition of the response to 40 mM KCl (Figure 6b) when added at the same time points. However, the antivenom, when added after 60 min, did not prevent the 10 µg/mL *P. australis* venom-mediated inhibition of direct twitches or contractile response to 40 mM KCl (Figure 6a,b). In contrast, the physical removal of the venom by washing the bath with physiological salt solution 5 min after adding the venom partially prevented the 10 µg/mL *P. australis* venom-mediated inhibition of direct twitches but not the venom-mediated inhibition of the contractile response to 40 mM KCl (Figure 6c,d). Washing after 30 min and 60 min did not prevent the *P. australis* venom-mediated inhibition of direct twitches or inhibition of the contractile response to 40 mM KCl (Figure 6c,d).

## 3. Discussion

We have shown that both Sri Lankan Russell’s viper and Mulga snake venoms are myotoxic in the avian skeletal muscle with the latter showing more potent myotoxicity. The myotoxicity induced by Sri Lankan Russell’s viper venom was fully neutralised by Bharat antivenom, partially neutralised by Vins antivenom and partially cross-neutralised by the Australian polyvalent antivenom when antivenoms were added before venom (i.e., pre-venom prevention study). Myotoxicity induced by Mulga snake venom was fully neutralised by Australian polyvalent antivenom, partially cross-neutralised by Vins antivenom but not cross neutralised by Bharat antivenom when antivenoms were added before venom. Myotoxicity induced by both Russell’s viper and the Mulga venom was only partially prevented by antivenoms raised against the specific venoms when added 5 min after the venom (i.e., post-venom prevention study). As the time period between the addition of venom and the addition of antivenom was increased, the efficacy of the antivenoms gradually decreased to almost nil at 60 min.

The myotoxicity induced by both Russell’s viper and Australian Mulga snake venoms was fully prevented by Bharat and Australian polyvalent antivenoms when added in manufacturer-recommended concentrations before the venom, indicating that both antivenoms have adequate antibody coverage for the myotoxins in both venoms. However, the failure of both antivenoms to fully prevent myotoxicity when added only 5 min after the venom indicates that the action of the myotoxins in both venoms is rapid and irreversible, once the myotoxic changes are initiated. Therefore, it appears as though the antivenoms can prevent further muscle damage but are unable to reverse myotoxicity. This may suggest that the F(ab’)_2_ antibody fragments (~110 kDa) are unable to reach the target sites as quickly as most of the PLA_2_ myotoxins (~15 kDa), which are much smaller in size. Alternatively, the binding of the antibody fragments to the antigenic sites of the toxins already at the target sites fails to prevent the toxin-target interactions, or the time period required for the antivenom to bind to venom is longer than the shorter time period required for the toxins to cause irreversible damage [2,12]. The efficacy of Indian polyvalent antivenom and Australian polyvalent antivenoms on already initiated Russell’s viper venom- and Mulga venom-induced myotoxicity was comparable to the action of physical removal of the toxins, by washing the bath, at the same time points. This indicates that the action of the antivenoms on the venom-induced myotoxicity is likely to be based on trapping the toxins and steric hindrance, which does not allow them to reach target sites. Therefore, the use of synthetic small-molecular toxin inhibitors with higher tissue penetration potential, such as Varespladib (LY315920), as an adjunct therapy to antivenom for systemic myotoxicity, should be further explored [13].

Myotoxicity induced by both venoms was at least partially preventable (or reversible) by the antivenoms raised against each of these venoms up to 1 h after the addition of venom, indicating that the therapeutic window of opportunity is likely to be longer for Russell’s viper and Mulga venom-induced myotoxicity. This longer window of opportunity for PLA_2_ myotoxin-rich Russell’s viper and Mulga snake venom-induced myotoxicity contrasts with our previous observations on the myotoxicity induced by the cytotoxin-rich Indian cobra venom, which shows a much narrower window of opportunity for the antivenoms to prevent further muscle injury [14]. This difference may be due to the differences between the mechanism of actions of the cytotoxins and PLA_2_ myotoxins, as well as the fact that cytotoxins are smaller (<8 kDa) hence able to reach target sites more quickly than the PLA_2_ myotoxins (~15 kDa). However, it should be noted that the present study does not represent the pharmacokinetics of venom in human envenoming. In human envenoming, the venom toxins are injected intramuscularly or subcutaneously and enter the circulation before reaching distant muscle cells. The antivenom is given intravenously, allowing direct access to the circulation. Therefore, the window of opportunity for antivenoms to prevent venom-induced myotoxicity is likely to be considerably longer in human envenoming. This is consistent with the clinical observations in which myotoxicity in Mulga snake envenomed patients was prevented when antivenom was given within 2 h of the bite, despite the patients being severely envenomed [8].

The chick-biventer cervicis model is a well-established model for testing the myotoxic effects of snake venom [4,15]. The model ensures the selective monitoring of myotoxicity, by direct electrical stimulation of the muscle belly as well as chemically (with the depolarising agent KCl) while negating any nerve-mediated responses by using the nicotinic receptor antagonist d-tubocurarine. The utility of this model in examining antivenom efficacy with regards to the myotoxicity of snake venom has been largely explored using a pre-venom prevention approach, i.e., the test antivenom is added prior to the test venom. This process determines whether the antivenom contains antibodies, or their fragments, which bind with the myotoxins in a manner that prevents the binding of myotoxin to the target sites. However, this scenario does not approximate the real-life situation in which patients receive antivenom potentially hours after the snakebite. To utilise the chick-biventer cervices model to more closely approximate the real-life scenario of snakebite, we followed a post-venom approach in which the antivenom was added to the bath at different time points after the venom. However, this approach forces the muscle, which has already been exposed to venom, to rapidly adapt to new bath conditions, such as rapid changes in the physical or chemical environment, with the addition of antivenom or replacing the buffer in the bath. In our post-venom prevention experiments, we observed either increase or rapid reduction of direct twitches in the immediate aftermath of the addition of antivenoms and washing. The twitches of the muscles gradually settled within the three-hour observation period and based on the response of the muscles to KCl, any muscle injury caused by antivenom on its own, or washing alone, could be excluded.

## 4. Conclusions

The myotoxicity of Sri Lankan Russell’s viper and Mulga snake venoms was prevented by homologous antivenoms when added to the bath before the venom but was only partially prevented when antivenoms were added 5 min after the venom, with the prevention becoming poorer when the delay between venom and antivenom increased. The action of antivenom was comparable to the physical removal of toxins from the site of action. This indicates that the myotoxicity is preventable only if the toxins are neutralised before they reach their target sites, and the role of antivenom is likely to trap the toxins or steric hindrance within the circulation, not allowing the toxins to reach target sites in muscles.

## 5. Material and Methods

### 5.1. Venoms

Desiccated and crystallised Sri Lankan Russell’s viper venom (*Daboia russelii*) from Sri Lanka and freeze-dried pooled Australian Mulga snake venom (*Pseudechis australis*) from Australia were used for this study. Venom was dissolved in distilled water and stored at −80 °C.

### 5.2. Antivenoms

Indian polyvalent antivenoms manufactured by Vins Bioproducts Limited (Telangana, India; Batch No: 01AS17008; date of expiry: March 2021) and Bharat Serums and Vaccines Limited (Maharashtra, India; Batch No: A05317042; date of expiry: January 2021) were used for this study. The antivenoms were reconstituted in 2 mL of distilled water instead of the 10 mL recommended by the manufacturer to minimise the final volume of the antivenom solution to be added into the organ baths. According to the manufacturer, 1/10 of the content of a single antivenom vial (0.2 mL of the antivenom solution made for the present study) neutralises 0.6 mg of Sri Lankan Russell’s viper venom.

Australian polyvalent antivenom (equine) (Seqirus, Parkville, VIC, Australia; Batch No: 055519001; date of expiry: October 2017) was used for this study. Australian antivenoms come in liquid form and, according to the manufacturer’s instructions, this vial of polyvalent antivenom contained 18,000 units of *P. australis* antivenom in 48.54 mL. In all experiments, the required antivenom amount to neutralise the venom amount in the organ bath was calculated based on the above recommendation by the manufacturer. The experiments were conducted after the expiry of the Australian polyvalent antivenom. It has been shown that antivenoms, including some Australian, North and South American as well as African antivenoms do not significantly lose their activity several years after the date of expiry and show significant efficacy even after 20 years of their expiration date [16,17]

For cross-neutralisation experiments, a heterologous antivenom amount similar to the required homologous antivenom amount was used.

### 5.3. Chick-Biventer Cervicis Nerve-Muscle Preparation

Male chickens (aged 4–10 days) were humanely killed by exsanguination following CO_2_ inhalation. Biventer cervicis nerve-muscle preparations were dissected out and then mounted on wire tissue holders under 1 g resting tension in 25 mL organ baths. The tissues were maintained at 34 °C and bubbled with 95% O_2_ and 5% CO_2_ in a physiological salt solution of the following composition (mM); 118.4 of NaCl, 4.7 of KCl, 1.2 of MgSO_4_, 1.2 of KH_2_PO_4_, 2.5 of CaCl_2_, 25 of NaHCO_3_ and 11.1 of glucose. Direct twitches were evoked by stimulating the muscle belly (rate: 0.1 Hz; pulse duration: 2 ms) at a supramaximal voltage (20–30 V) with the two electrode loops directly encircling the muscle belly, using an electronic stimulator (ADInstruments Pty Ltd., Bella Vista, NSW, Australia). Contractile responses of the tissues to KCl (40 mM for 30 s) were obtained in the absence of muscle stimulation. Following that, direct stimulation of the muscle was resumed. The preparations were stimulated for 10 min before the addition of venom or control (physiological salt solution). In antivenom prevention experiments, antivenom was added to the bath after 10 min of direct stimulation of the tissues, and the venom was added once the tissues were equilibrated in antivenom for 10 min. After adding venom, washing or the addition of antivenom was practised at different time intervals. All experiments were run for 3 h. At the conclusion of the experiment, KCl was re-added as above.

### 5.4. Data Analysis and Statistics

Direct twitch responses and responses to KCl were measured via an MLT0201 force transducer (ADInstruments Pty Ltd., Bella Vista, NSW, Australia) and recorded on a PowerLab system (ADInstruments Pty Ltd., Bella Vista, NSW, Australia). Responses were expressed as the percentages of their pre-venom values. To compare the pre-and post-experiment responses to KCl, one-way ANOVA was used followed by Tukey’s multiple comparison post-tests. Data are presented in the form of the mean +/− standard error of the mean (SEM) of three to five experiments. Full neutralisation of myotoxicity was defined as the antivenom leading to a recovery of both the direct twitches and the response of the muscles to 40 mM, to an extent not different from the controls. Partial neutralisation was defined as the antivenom leading to a recovery of the direct twitches of muscles and/or the response of the muscles to 40 mM, to an extent that is different from the controls as well as the venom. The absence of neutralisation was defined as both the recovery of the direct twitches of muscles and/or the response of the muscles to 40 mM were different from the controls and not different from the venom.

All statistical analyses and the presentation of data were generated using GraphPad Prism 9.0.1 software (GraphPad Software Inc., La Jolla, CA, USA). For all statistical tests, *p* < 0.05 was considered statistically significant.

## Data Availability

Not applicable.
